# Predicting *Corynebacterium glutamicum* promoters based on novel feature descriptor and feature selection technique

**DOI:** 10.3389/fmicb.2023.1141227

**Published:** 2023-03-02

**Authors:** HongFei Li, Jingyu Zhang, Yuming Zhao, Wen Yang

**Affiliations:** ^1^College of Life Science, Northeast Forestry University, Harbin, China; ^2^College of Information and Computer Engineering, Northeast Forestry University, Harbin, China; ^3^Department of Neurology, The Fourth Affiliated Hospital of Harbin Medical University, Harbin, China; ^4^International Medical Center, Shenzhen University General Hospital, Shenzhen, China

**Keywords:** promoter, *Corynebacterium glutamicum*, physicochemical properties, analysis of variance, hierarchical clustering, feature selection, random forest

## Abstract

The promoter is an important noncoding DNA regulatory element, which combines with RNA polymerase to activate the expression of downstream genes. In industry, artificial arginine is mainly synthesized by *Corynebacterium glutamicum*. Replication of specific promoter regions can increase arginine production. Therefore, it is necessary to accurately locate the promoter in *C. glutamicum*. In the wet experiment, promoter identification depends on sigma factors and DNA splicing technology, this is a laborious job. To quickly and conveniently identify the promoters in *C. glutamicum*, we have developed a method based on novel feature representation and feature selection to complete this task, describing the DNA sequences through statistical parameters of multiple physicochemical properties, filtering redundant features by combining analysis of variance and hierarchical clustering, the prediction accuracy of the which is as high as 91.6%, the sensitivity of 91.9% can effectively identify promoters, and the specificity of 91.2% can accurately identify non-promoters. In addition, our model can correctly identify 181 promoters and 174 non-promoters among 400 independent samples, which proves that the developed prediction model has excellent robustness.

## 1. Introduction

*Corynebacterium glutamicum* is a prokaryote, which was first discovered in the 1950s (Sano, 2009). It is mainly responsible for the production of L-glutamic acid and has played a huge potential in the production of amino acids in the industrial field. *C. glutamicum* is considered the best bio-manufacturing substrates by many countries because it can produce amino acids with few nutrients and sufficient capacity (Sun et al., 2011; Vertes et al., 2012). Considering the excellent characteristics of *C. glutamicum*, the genome has been modified to produce a variety of amino acids, organic acids, alcohols, and proteins through biological genetic technology (Okino et al., 2008; Hu et al., 2013). At the beginning of the 20th century, *C. glutamicum* first was published its complete genome sequence, named *C. glutamicum* ATCC 13032. The whole genome consists of a circular chromatin with a length of 3282708 bp, containing 3000 coding protein genes, and the ‘C + G’ content is 53.8% (Kalinowski et al., 2003). The complete genome sequencing of this species provides convenient conditions for gene editing and regulatory analysis that can further improve the efficiency of *C. glutamicum* to produce amino acids (Barrangou and Horvath, 2017; Cho et al., 2017; Jiang et al., 2017; Huang et al., 2019). The above biotechnology mainly involves the knockout and inactivation of specific genes, and the key is to locate the starting site of genes and the promoter region of the target gene (Okino et al., 2008; Theron and Reid, 2011; Silar et al., 2016). In Hebert et al. (2018) and Shang et al. (2018) designed a special promoter, which improved the expression level of sucCD and the production of L-lysine. Thus, it is very important to identify and locate the promoter of *C. glutamicum*.

The promoter, as a pivotal regulatory element, is responsible for activating the expression of target genes (Canzio et al., 2019; Xiao et al., 2019; Jeon and Tucker-Kellogg, 2020). In preparation for gene expression, promoters are affected by macromolecular complexes that are produced by the combination of specific transcription factors and regulatory factors to complete the transcription from DNA to RNA (La Fleur et al., 2022; Liu et al., 2022; Rengachari et al., 2022). In industrial systems, the recognition of promoters of *C. glutamicum* requires the help of Sigma factors, which requires the support of gene isolation, polymerase chain reaction (PCR), and gene cloning techniques (Blumenstein et al., 2022; Stepanek et al., 2022). Although the wet lab methods described above can specifically identify promoters, they are time - and labor-consuming, and it is essential to develop a method-based calculating model to rapidly identify promoters. At present, models of promoter recognition already exist for many species (Silar et al., 2016; Bharanikumar et al., 2018; Leemans et al., 2019), but cannot be applied to Corynebacterium because of the large differences in homology between the species. Moreover, these models employed features that do not accurately describe the inherent properties of DNA sequences, resulting in poor overall prediction performance. For example, in the human promoter recognition task, Li et al. (2022b) used five feature descriptors to express DNA sequences, but the final prediction accuracy was only 80%. Hence, it is necessary to design a mathematical prediction model to accurately identify the promoter of *C. glutamicum* for the industrial production of amino acids.

Here, we have collected promoter sequences that have been verified and annotated by experiments (Su et al., 2021), and designed a new feature expression method according to the distribution of multiple physical and chemical properties of sequence DNA. In addition, we have developed a novel feature selection method for redundant information between features. The proposed model has strong robustness by independent set verification.

## 2. Materials and methods

**Figure 1:**
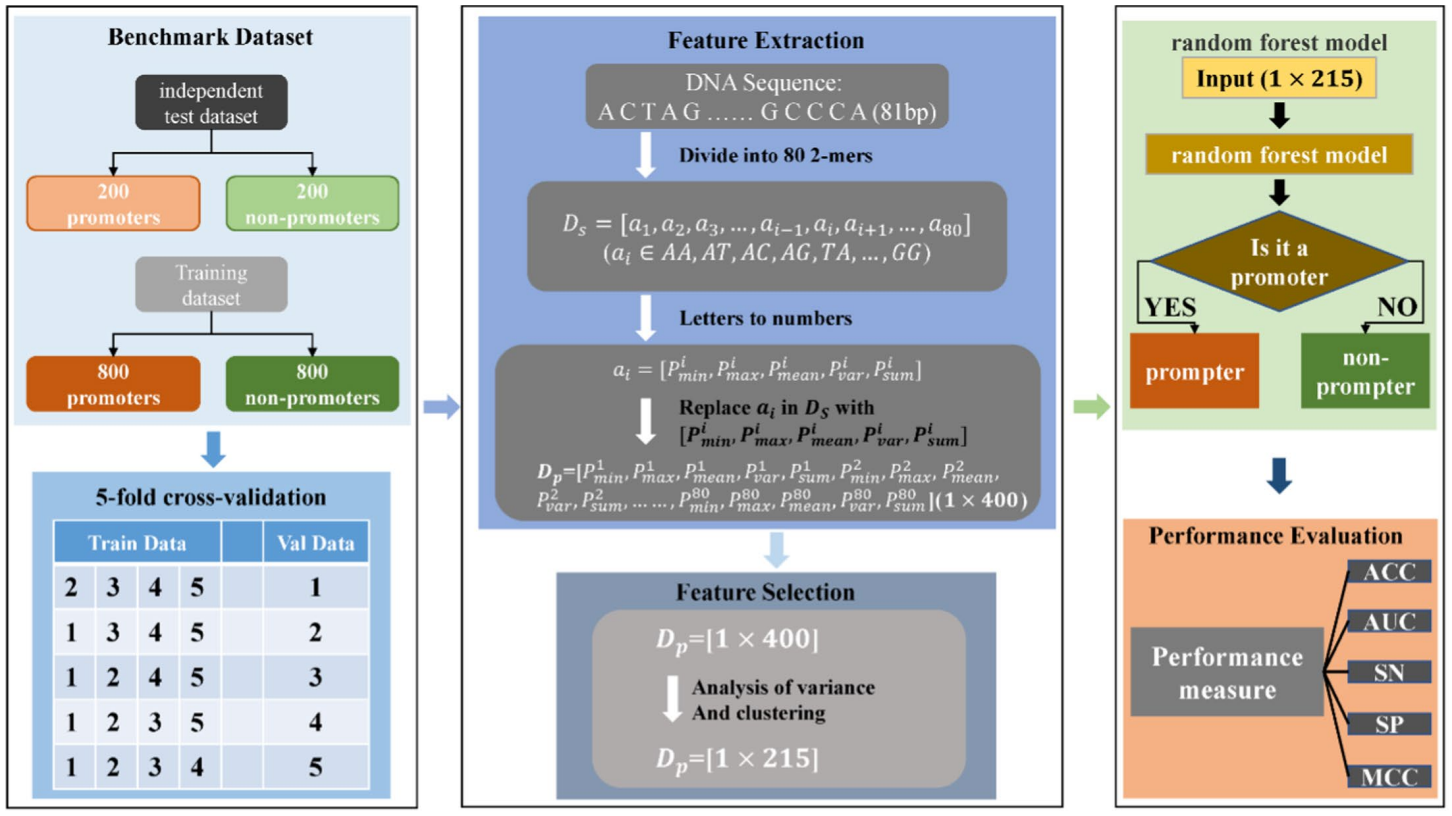
The workflow of *Corynebacterium glutamicum* promoter prediction model.

The following three conditions are indispensable to the excellent properties of the prediction model. First, building a rigorous and proven dataset. Second, designing the corresponding feature descriptor according to the inherent attributes of the sample and the specific distribution. Finally, selecting the machine learning algorithm that conforms to the regular pattern of descriptors. The flow of the whole method is drawn in Figure 1.

### 2.1. Benchmark dataset

To build a reasonable and interpretable dataset, the promoter of *C. glutamicum* selected comes from the PPD database that collected promoters of 63 eukaryotes, including 129,148 promoter sequences, each of which was confirmed by strict experiments (Su et al., 2021). Therefore, we take 3,581 promoters of *C. glutamicum* ATCC 13032 in the dataset as positive samples. Initially, we filter promoters with incomplete annotation information and the same starting site. Immediately, CD-HIT software was employed to reduce the sequence consisting of the filtered promoters to less than 0.6 (Li and Godzik, 2006; Huang et al., 2010). Finally, we obtained 1,000 promoter sequences with a length of 81 bp. For the selection of negative sample non-promoters, we downloaded the complete genome data from the GenBank database^1^, and randomly cut 81 bp from different gene fragments as the original negative samples to enhance the diversity of the sequence. Similarly, the CD-HIT was applied to reduce its sequence consistency to 60%, then we reserved 1,000 non-promoter sequences as negative samples. Aiming to prove the robustness of the model, 2000 samples are randomly divided into the training set and independent set according to the ratio of 8: 2, 800 positive samples and 800 negative samples were used for model fitting and training by five-fold cross-validation, and the remaining 200 positive samples and negative samples are employed to test the model’s ability to recognize the unlabeled sample.

### 2.2. Feature descriptor

**Figure 2:**
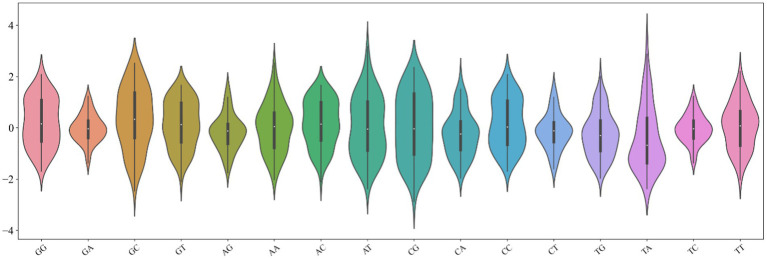
Violin chart of physical and chemical properties of 16 dinucleotides.

The key step in building a model is to accurately describe the inherent attributes and reflect the differences between samples. The combination of promoters with various regulatory elements is inseparable from the physicochemical properties of their bases, such as hydrophilicity and hydrophobicity. Therefore, we design a novel digital feature containing a variety of physical and chemical properties to describe the DNA sequence. First, we found the 90 physical and chemical properties of dinucleotides from published literature. Furthermore, we analyzed the distribution of these physicochemical properties of 16 dinucleotides (Dao et al., 2019). It can be found from Figure 2 that the distribution of 16 kinds of dinucleotides is more remarkable. The minimum value of dinucleotide ‘CG’ is obtained, while the maximum value of ‘TA’ is obtained. The ordinate of the violin chart corresponds to the frequency density of data distribution. For example, the distribution of ‘GA’, ‘CT’, and ‘TC’ shows a standard normal distribution, but their wave peaks and widths are different, so they have different mean values and variances. In addition, the area occupied by different dinucleotides also varies greatly, which infers the sum is diverse. Hence, we use the minimum, maximum, variance, mean, and sum of 90 physical and chemical properties to represent the overall physical and chemical property level of 16 dinucleotides, the 90 dimensional physical and chemical properties are replaced by 5 statistical parameters. The method can not only describe the distribution characteristics of dinucleotides but also greatly reduce the dimensions used to describe the descriptor. Suppose a DNA sequence s with length L, which can contain L-1 dinucleotides, as defined below:


(1) $$D_{s}=\left[a_{1},a_{2,}a_{3,}..,a_{i},..,a_{L-1}\right]\;\left(a_{i}\in AA,AT,AC,AG,TA\ldots ,GG\right)$$

where, *a*_*i*_ represents the arrangement of dinucleotides in the sequence, which is one of 16 kinds of dinucleotides because the four bases can form 16 kinds of arrangement combinations in pairs. Dinucleotide ai is converted into five statistical parameters, which are defined as follows:


(2) $$a_{i}=\left[p_{\min}^{i},\;p_{\max}^{i},\;p_{mean}^{i},\;p_{\mathrm{var}}^{i},\;p_{sum}^{i}\right]$$

where $p_{\min}^{i}$ , $p_{\max}^{i}$ , $p_{mean}^{i}$ , $p_{\mathrm{var}}^{i}$ , $p_{sum}^{i}$ is the minimum, maximum, mean, variance, and sum of 90 physical and chemical properties of the *i-*th dinucleotide. Therefore, the DNA sequence with a length of 81 bp is finally converted into an (81–1) × 5 = 400-dimensional feature vector. Detailed parameters of physical and chemical properties can be downloaded at http://lin-group.cn/server/iORI-PseKNC2.0/download.html.

### 2.3. Feature selection

Feature selection (Nasi et al., 2018; Zhang et al., 2019; Razzak et al., 2020) is to filter the redundant information in the original feature set to reduce the feature dimension and improve the calculation speed, which can reduce the model learning error caused by noise and improve (Aaron et al., 2019) the accuracy and robustness of the model. In the process of feature expression, 400-dimensional statistical parameters of physical and chemical properties are used to describe DNA sequences. Due to the similarity between multiple physical and chemical properties and dinucleotide distribution, it is necessary to apply a feature selection algorithm to eliminate highly similar features. Currently, the main feature selection algorithms employed in biological sequence recognition are analysis of variance (ANOVA) (UniProt Consortium, 2012; Hebert et al., 2018; Wu et al., 2020; Moorthy and Gandhi, 2021) and maximum relevance maximum distance (MRMD) (Zou et al., 2016; Ao et al., 2021). ANOVA mainly reflects the contribution of features to the model by calculating the difference between positive and negative samples, then features with less contribution are deleted. MRMD judges the independence between samples and labels through various distance formulas, and features with low independence are filtered. However, the above methods have some defects, ANOVA only measures the difference between positive and negative samples of features, without considering the similarity between features. Oppositely, MRMD lacks the characteristics of analysis of positive and negative samples.

**Figure 3:**
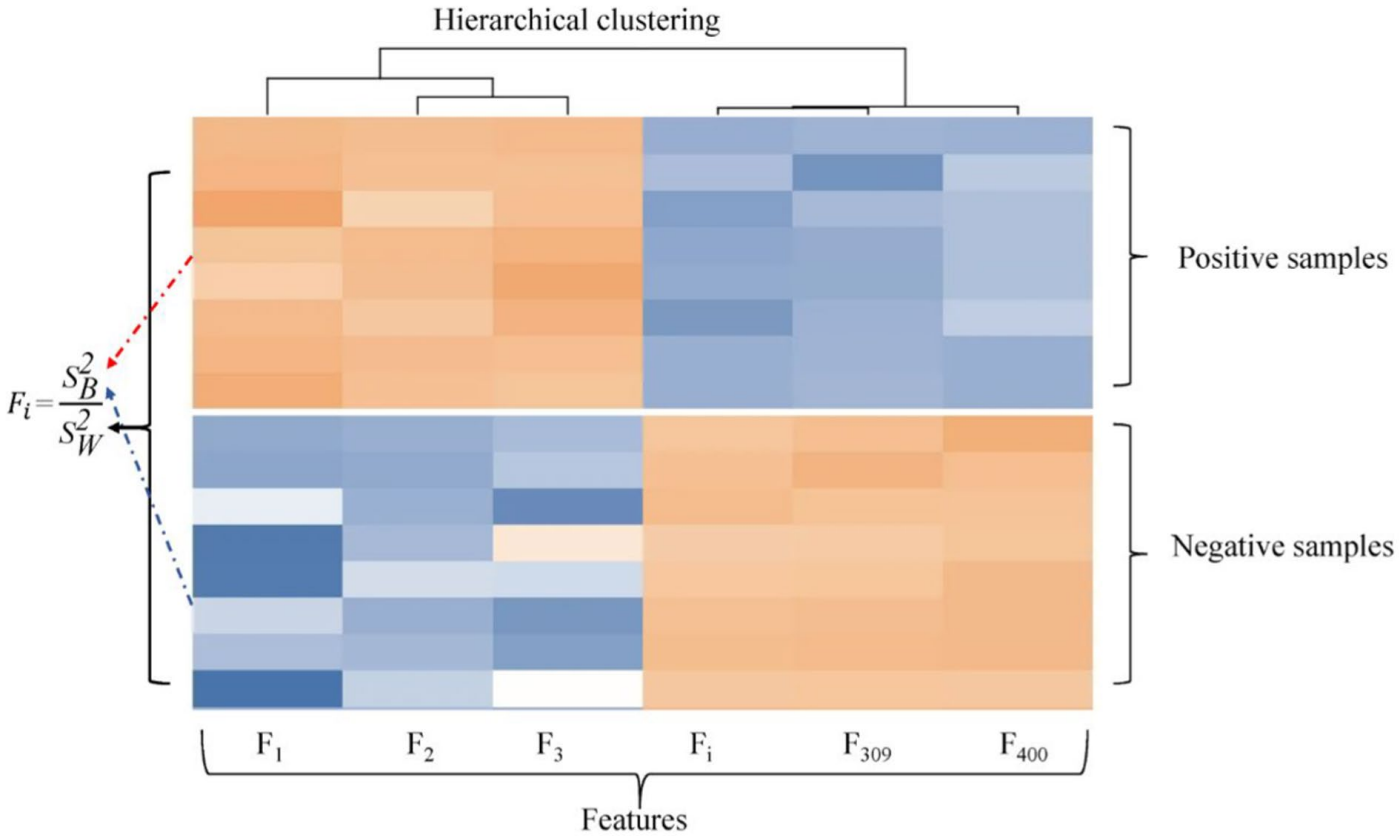
Feature Selection Schematic. *F_i_* is the *F* value of the *i*-th dimension feature, $S_{B}^{2}$ and $S_{W}^{2}$ are differences between groups and within groups.

Considering the advantages and disadvantages of MRMD and ANOVA, we propose a novel feature selection method based on ANOVA and hierarchical clustering (HC) (Karna and Gibert, 2022; Zhu et al., 2022). As shown in Figure 3, the method comprehensively considers the similarity between features and the difference between a positive and negative sample of features. The first step is to calculate the *F* value of each one-dimensional feature, which is obtained by ANOVA of differences between groups and within groups, the ‘f_classif’ function in the ‘sklearn’ Python package is used to calculate the F value of each dimension feature. The second step is the hierarchical clustering analysis of features, the ‘AgglomerativeClustering’ function in ‘sklearn’ Python package is employed to measure the similarity between features. This algorithm mainly classifies two pairs of features into one cluster according to the distance between features, and we reserve the features with a large *F* value in each cluster of the first-level clustering results, when the *F* values are the same, a feature was selected at random. As shown in Figure 3, in the first-level clustering results, *F*_2_ and *F*_3_ are clustered into one cluster. If *F*_2_ is larger than *F*_3_, the feature of *F*_2_ is retained, while *F*_1_ is directly retained for a cluster alone. Therefore, the 3 dimensions feature ultimately remains 2 dimensions feature. In practical application, the 400 dimensions features are selected as the best subset of 215 dimensions for the final model construction.

### 2.4. Model development

The construction of the prediction model is the process of fitting sample labels according to the distribution of features. Because the feature descriptor designed is based on statistical parameters, it can be seen from Figure 2 that the designed feature distributions are very different, the positive and negative samples of feature subsets after feature selection also have this property. Therefore, the promoter prediction model has superior performance that required to accurately measure the confusion between sample features. The RF algorithm distinguishes the category of samples according to the confusion of feature information, so the algorithm is applied to the construction of the classifier. RF judges the disorder degree of samples according to the ‘Gini’ coefficient. A small ‘Gini’ coefficient means that the lower the disorder degree of samples, the greater the probability of correct recognition. The ‘RandomForestClassifier’ function in the ‘sklearn’ Python package is used to build the model. In the process of model training, the value range of five parameters is mainly adjusted by grid searching, the ‘n_estimators’ is 80 to 150 with 5 steps, the ‘max_depth’ is 15 to 20 with 1 in step, ‘min_samples_leaf’ is 1 to 8 with 1 in step, ‘min_samples_split’ is 2 to 5 with 1 in step, and ‘max_features’ is 0.1 to 1 with 0.1 in step, respectively. The determination of the best combination parameters is based on five-fold cross-validation.

### 2.5. Evaluation parameters

The performance of the model needs to be evaluated by some indicators. For the second classification problem, the most common evaluation parameters (Xu et al., 2018; Chao et al., 2019; Demidova, 2021; Li et al., 2022a,b) are sensitivity (Sn), specificity (Sp), accuracy (Acc), Matthews correlation coefficient (MCC) and area under the Receiver Operating Characteristic (ROC) curve (AUC), which are defined as follows:


(3) $$\{\begin{array}{l} Sn=\frac{TP}{TP+FN}\\ Sp=\frac{TN}{TN+FP}\\ Acc=\frac{TP+TN}{TP+FP+TN+FN}\\ MCC=\frac{\left(TP\times TN\right)-\left(FP\times FN\right)}{\sqrt{\left(TP+FN\right)\left(TN+FP\right)\left(TP+FP\right)\left(TN+FN\right)}} \end{array}$$

where TP and FP are correctly labeled promoters and incorrectly labeled promoters, and TN and *F* are correctly labeled non-promoters and incorrectly labeled non-promoters. Sn is employed to describe the model’s ability to detect promoters, while Sp is employed to describe non-promoters. Acc, MCC, and AUC are used to describe the overall prediction capability of the model.

## 3. Result and discussion

### 3.1. Model performance analysis

**Figure 4:**
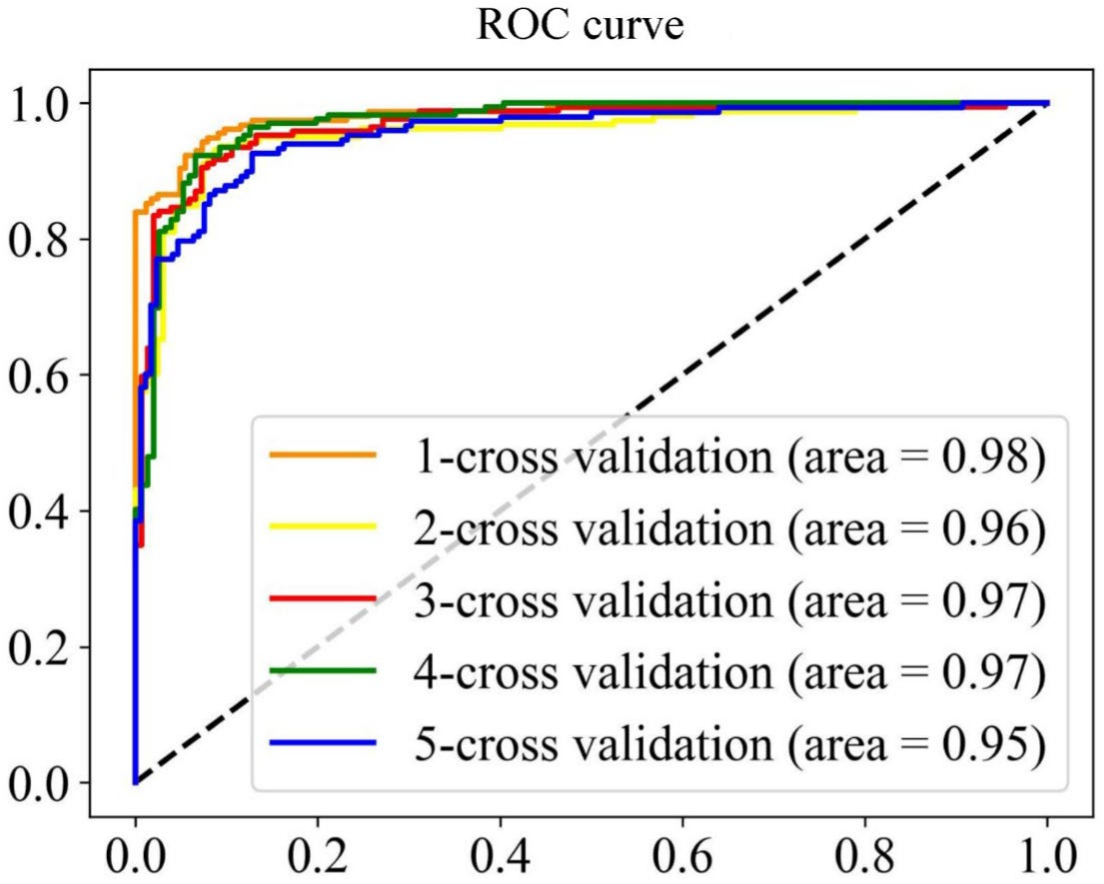
ROC curve of cross validation results.

A model with superior performance can not only accurately fit the sample labels on the training set, but also accurately judge the labels of unknown samples. To prove that the model proposed has the above qualifications, we summarize the results of five-fold cross-validation and independent set validation based on the RF (Zhang et al., 2009; Wei et al., 2017; Ao et al., 2021) prediction model in Table 1. It can be found from the table that in the first cross-validation, Sn, Acc and MCC, respectively, obtained the maximum value of 94.51, 93.13, and 86.26%, and Sp obtained the maximum value of 93.49% at the fourth cross-validation, which shows that different partition strategies of the dataset affect the fitting of the model, so the mean value of five-fold cross-validation is finally regarded as the standard prediction result. In general, the model proposed can accurately identify promoters and non-promoters, with an average Acc of 91.56%, Sn of 91.87%, and Sp of 91.17%. In addition, it can be seen from the ROC curve in Figure 4 that the performance of the model is superior, which shows that the AUC reaches more than 95%. To verify the robustness of the model, we conducted independent set tests and found that the model can also accurately distinguish promoters and non-promoters. In 400 independent samples, the model can correctly identify 181 promoters and 174 non-promoters, which confirms that our proposed model is capable of predicting annotated promoter fragments.

**Table 1:** The prediction performance of different subsets in RF.

Descriptor	Sn (%)	Sp (%)	Acc (%)	MCC (%)
1-th validation	**94.51**	91.67	**93.13**	**86.26**
2-th validation	92.59	91.39	91.88	83.75
3-th validation	91.39	91.72	91.56	83.08
4-th validation	90.73	**93.49**	92.19	84.32
5-th validation	90.12	87.84	89.06	77.99
Mean of validation	91.87	91.17	91.56	83.08
Independent verification	90.50	87.00	88.75	77.55

The bold value represents the maximum value. Sn, sensitivity; Sp, specificity; Acc, accuracy; MCC, matthew correlation coefficient.

### 3.2. Feature composition analysis

**Figure 5:**
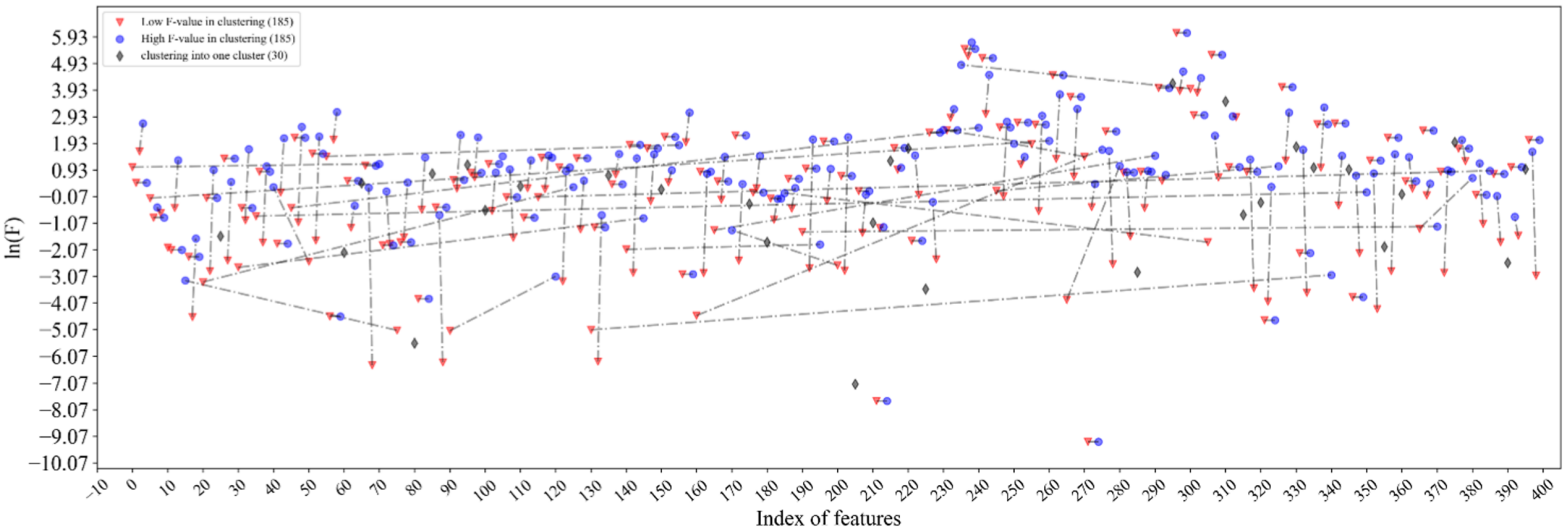
Visualization of feature selection results. The features marked in red and marked in blue are clustered together and connected by dotted lines. The black diamond indicates that the samples are grouped into a single category.

The excellent performance of the proposed model is driven by the accurate representation of feature descriptors and the filtering of redundant information by feature selection. It can be seen from Figure 5 that the features marked in red and marked in blue are clustered together and connected by dotted lines. The connected red-blue paired samples have high similarity, and the red samples with low *F* values are removed for noise removal, which horizontal dashed lines represent the points with far distance for dimensions, while vertical dashed lines represent the points with close distance, which proves that our method can filter global features rather than local features. Hence, 370 features are filtered out in half. The black diamond indicates that the samples are grouped into a single category, and they are directly retained. Finally, the feature dimension used to construct the samples is 215. More importantly, the feature accuracy of 400 dimensions has been improved from 90.69 to 91.56% of 215 dimensions, which shows that our feature selection method based on ANOVA and HC can reduce the redundancy of features and improve the model performance to a certain extent.

### 3.3. Multi-algorithm analysis

In the process of building the model, the RF classification algorithm is selected according to the characteristics of descriptor distribution. Although this algorithm has achieved good prediction performance, it is still possible that other classification algorithms have better results, such as K nearest neighbor (KNN) (Wang et al., 2012; Demidova, 2021), Support vector machine (SVM) (Xu et al., 2018; Xiao et al., 2019), Multi-layer perceptron (MLP) (Majidzadeh Gorjani et al., 2021; Lin et al., 2022). Therefore, we compared different classification algorithms based on filtered features. It can be seen from Table 2 that in cross-validation, the performance of the RF is the best. The prediction accuracy of SVM is 87.63%, which is closest to the RF, followed by the MLP with an accuracy of 85%, and the worst KNN accuracy is only 75.62%. The situation of independent verification is consistent with the above situation. And only the accuracy of the RF algorithm has the smallest difference between independent set verification and cross verification, which also proves that the proposed model has strong robustness and small overfitting analysis.

**Table 2:** Comparison of different classification algorithms.

Classifier	Verification	*Sn* (%)	*Sp* (%)	*Acc* (%)	*MCC* (%)
KNN	Five-fold cross-validation	72.98	78.55	75.62	51.58
	Independent testing	67.00	81.00	74.00	48.48
SVM	Five-fold cross-validation	88.59	86.77	87.63	75.31
	Independent testing	82.00	81.50	81.75	63.50
MLP	Five-fold cross-validation	85.25	85.58	85.44	70.85
	Independent testing	79.00	82.50	80.75	61.51
RF	Five-fold cross-validation	**91.87**	**91.17**	**91.56**	**83.08**
	Independent testing	90.50	87.00	88.75	77.55

The bold value represents the maximum value. Sn, sensitivity; Sp, specificity; Acc, accuracy; MCC, matthew correlation coefficient; SVM, support vector machine; RF, random forest; MLP, multi-layer perceptron; KNN, k-nearest neighbors.

## 4. Conclusion

In this work, we collected promoter and non-promoter sequences of *C. glutamicum* with annotation information, then designed a feature descriptor based on statistical parameters according to the distribution characteristics of physical and chemical properties. Further, we defined the novel feature selection method to filter redundant information among features. Finally, we successfully built the prediction model based on RF that can accurately identify promoters. In a word, the model we designed can accurately identify the promoter sequences of eukaryotes, and we hope that the feature descriptors and feature selection methods designed can s make positive contributions to other sequence classification problems.

## Data availability statement

The original datasets and code used in this study can be found at https://github.com/Hongfeipower/Predicting-Cornebacterium-glutamicum-Promoters.

## Author contributions

HL and YZ designed the study. HL and JZ carried out all data collection and drafted the manuscript. WY and YZ revised the manuscript. All authors contributed to the article and approved the submitted version.

## Funding

This work has been partially supported by the National Natural Science Foundation of China (61971119, 62272094), and the National Key R&D Program of China (2021YFC2100103).

## Conflict of interest

The authors declare that the research was conducted in the absence of any commercial or financial relationships that could be construed as a potential conflict of interest.

## Publisher’s note

All claims expressed in this article are solely those of the authors and do not necessarily represent those of their affiliated organizations, or those of the publisher, the editors and the reviewers. Any product that may be evaluated in this article, or claim that may be made by its manufacturer, is not guaranteed or endorsed by the publisher.

## Footnotes

^1^ https://www.ncbi.nlm.nih.gov/nuccore/NC_006958.1

## Abbreviations

SVM: support vector machine
RF: random forest
MLP: multi-layer perceptron
KNN: k-nearest neighbors
Sn: sensitivity
Sp: specificity
Acc: accuracy
MCC: matthew correlation coefficient
ROC: receiver operating characteristic
AUC: area under receiver operating characteristic (ROC) curve
ANOVA: analysis of various
HC: hierarchical clustering
